# Open System Tribology and Influence of Weather Condition

**DOI:** 10.1038/srep32455

**Published:** 2016-08-30

**Authors:** Yezhe Lyu, Ellen Bergseth, Ulf Olofsson

**Affiliations:** 1 Department of Machine Design, (KTH) Royal Institute of Technology, SE-100 44 Stockholm, Sweden

## Abstract

The tribology of an open system at temperatures ranging between 3 °C and −35 °C, with and without snow, was investigated using a pin-on-disc tribometer mounted in a temperature-controlled environmental chamber. The relationship between the microstructure and ductility of the materials and the tribology at the contacting surfaces was investigated. The study shows that during continuous sliding, pressure causes snow particles to melt into a liquid-like layer, encouraging the generation of oxide flakes on the contact path. The friction coefficient and wear rate are dramatically reduced through an oxidative friction and wear mechanism. In the absence of snow, the tribological process is controlled by the low temperature brittleness of steel in the temperature range from 3 °C to −15 °C. At these temperatures, cracks are prone to form and extend on the worn surfaces, resulting in the spalling of bulk scraps, which are crushed into debris that increases the friction coefficient and wear rate due to strong abrasion. When the temperature falls to −25 °C, an ice layer condenses on the metal surfaces and relaxes the tribological process in the same way as the added snow particles, which significantly decreases the friction and wear.

Tribology is important in systems that contain interacting surfaces in relative motion, such as bearings, gears, and brakes. The interacting surfaces may have very different characteristics as regards roughness, hardness, plasticity, brittleness and diverse contaminants, all of which significantly influence the tribological process^1^. While some of these factors are determined during the manufacturing process, others vary with the operating environment. This environment may be closed or open. Closed systems (such as the seals, valves and gears in a gearbox) are theoretically isolated from the natural environment, and so are not affected by weather conditions. Open systems on the other hand, are greatly affected by weather conditions such as precipitation, temperature and humidity – feet, for example, may slip when it is raining, and tires may spin on a snowy road. Temperature influences the tribological process by affecting the properties of the interacting surfaces. Polymers, for example, are harder at low temperatures than at room temperature^2^, and body-centred cubic steel experiences a ductile-to-brittle transition with decreasing temperature^3^. All these changes in properties affect the tribological process at the molecular scale when the interacting surfaces move relative to one another^4^. Therefore, even the simplest tribological process may exhibit great complexity, such as local deformation and corrosive processes^5^,^6^,^7^,^8^,^9^.

Tribology at the wheel-rail contact plays a key role in railway performance. Friction controls the tracking and braking, while wear affects reliability and endurance. Researchers have investigated the effect of temperature, humidity and natural contaminants (e.g. sand, water, and leaves) on tribology at the wheel-rail contact^10^,^11^,^12^,^13^,^14^. However, more research is needed on wheel-rail tribology in winter when the temperature is below zero and snow particles are present. The reason for the lack of research in this area is the difficulty of achieving stable control of sub-zero temperatures. Although several apparatuses have been set up to obtain a cryogenic environment covering an extensive temperature range (−268 °C to 20 °C)^15^,^16^,^17^, all of them ignore the influence of natural air and contaminants as they all achieve the low temperature by dint of a vacuum or liquid cryogen atmosphere. An open system such as a wheel-rail system usually operates in a habitable environment.

Most countries at high latitudes experience problems with wheel-rail contact in winter^18^,^19^,^20^,^21^. One possible reason is the ductile-brittle transition that wheel and rail steels experience as the temperature drops. The impact energy of a typical grade rail steel (UIC60 900A) was tested and the results are shown in Fig. 1. The ductile-brittle transition can be seen as the temperature decreases from 20 °C to −35 °C. As the rail becomes more brittle, the risk of crack generation increases, which may accelerate wear damage and cause traffic to be halted. Another possible reason is that snow dragged into the wheel-rail contact acts as a lubricant, resulting in loss of adhesion. Thus these two factors need to be investigated systematically.

For the current project, a sizable quasi-sealed and temperature-controlled environmental chamber was built to simulate cold weather in the real world. The sliding contact was simulated using a pin-on-disc tester. Much effort was devoted to investigating the tribological performance between the simulated wheel-rail contact at different temperatures with and without snow. The combination of contact pressure and sliding speed represented a common rail head-wheel tread contact condition. The range of temperatures tested covered the typical temperature range in high latitude countries. The microstructure of the metal and worn features were analysed by scanning electron microscopy (SEM) and energy dispersive X-ray spectroscopy (EDS). Wear was examined using a Talysurf roughness tester. On the basis of the experimental results, deductions were made about the influence of varying environmental temperatures on the tribological performance at the wheel-rail contact. A possible pattern of behaviour of the snow particles at the wheel-rail contact is also proposed.

## Results

### Tribological performance of the pins

**Figure 2:**
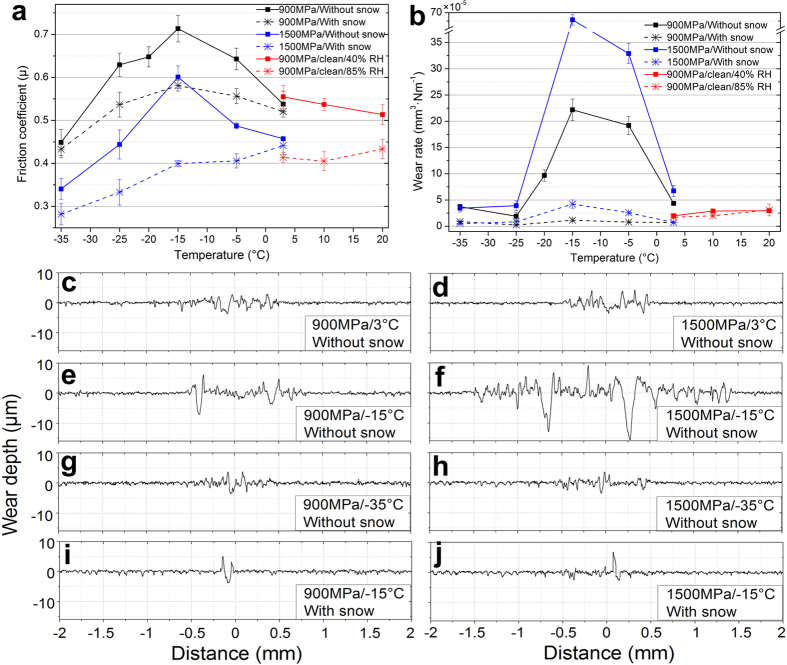
Results (mean value and standard deviation) of the (**a**) friction coefficient and (**b**) wear rate on the pins as a function of temperature (the results from −35 °C to 3 °C in black and blue are from the current research project and the results from 3 °C to 20 °C in red are cited from a previous study^13^ for comparison). (**c**–**j**): Wear track profiles of discs tested at two loads and different temperatures with or without snow addition.

Figure 2a,b show the mean value with error bars corresponding to the standard deviations of the friction coefficient and wear rate on the pins as a function of temperature. The results shown in black and blue were obtained in the current study (temperature ranges from −35 °C to 3 °C), and the results in red were obtained in a previous study (3 °C to 20 °C)^13^. It can be seen that the friction coefficient and wear rate remain quite stable in the temperature range from 3 °C to 20 °C. However, in the range from −35 °C to 3 °C, the friction coefficient and wear rate show strong temperature dependence.

For the current study, the friction and wear rate overall showed a decrease when snow particles were added into the contact at all test temperatures and both contact pressures. This decline was notable at −5 °C and −15 °C, where the friction coefficient decreased more than 0.1 when snow was added while the wear rate decreased one order of magnitude. The wear rate after snow was added decreased to a very low level, similar to that without snow (lower than 5 × 10^−5^ mm^3^·Nm^−1^) despite the change in temperature. The lowest friction coefficient and wear rate in the tests with snow added were observed at −35 °C for both contact pressures.

When snow was not added, friction and wear (solid lines in Fig. 2a,b) underwent a transition as a function of temperature. Initially the friction and wear increased as the temperature fell from 3 °C to −15 °C, and then sharply decreased with a further decrease in temperature from −15 °C to −35 °C. In the tests without snow, the friction coefficients obtained at 900 MPa at all test temperatures (black solid line in Fig. 2a) are consistently higher than those at 1500 MPa (blue solid line in Fig. 2a) at corresponding temperatures. The same phenomenon can be seen in the tests with snow added. The wear rates in tests at 900 MPa, on the other hand, are lower than those at 1500 MPa with and without snow.

### Measurement of wear tracks on the discs

The wear loss on the disc was too small to measure using an analytical balance. The width and depth of wear tracks were therefore used to evaluate the wear performance of the discs. Figure 2c–j present the wear track profiles on the discs tested under different conditions. These profiles were obtained with the help of a stylus instrument. It should be noticed that all the scales on the y-axis are centred and cover the range of −10 to 10 μm. On the x-axis the range is from −2 to 2 mm. Without snow, the widest and deepest wear tracks, representing the most severe wear condition on the disc, were obtained in the tests at −15 °C for both contact pressures. The narrowest wear track in the tests without snow occurred at −35 °C. Further, the widths and depths of the wear track on the disc tested with snow were much lower than those obtained without snow. All these findings are in accordance with the observation of the pins (Fig. 2a,b), demonstrating the concomitant wear conditions on the wheels and rails^22^.

## Discussion

Railway vehicles operate in a variety of climates, including sub-zero temperatures. This research revealed that friction and wear are much more sensitive to temperature between −35 °C and 3 °C than between 3 °C and 20 °C (Fig. 2).

### Lubrication effect of snow particles

**Figure 3:**
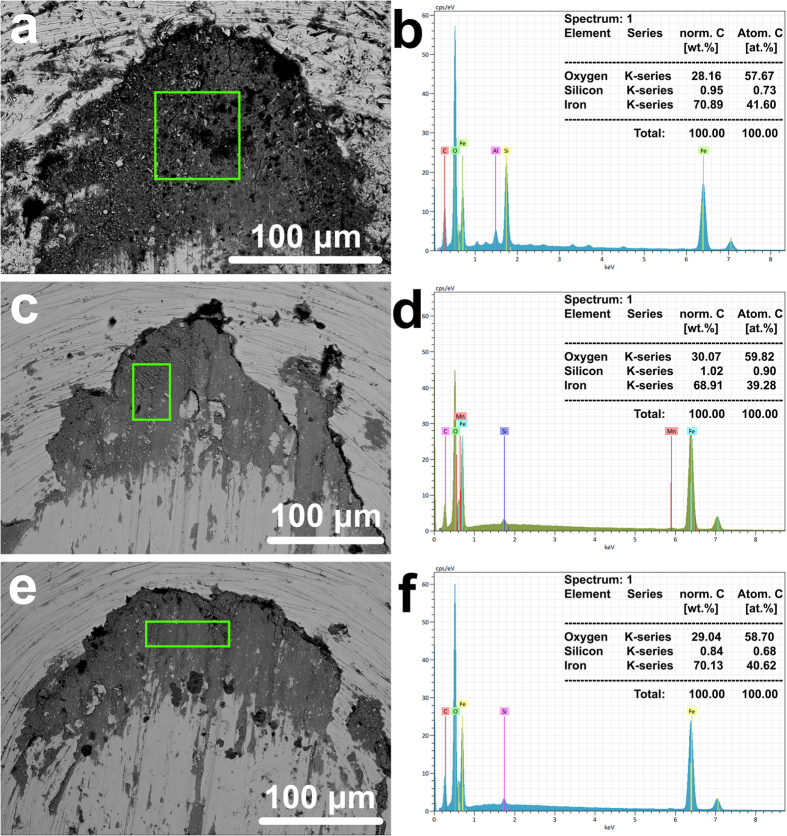
SEM micrographs of the oxide flakes on the worn surfaces of pins tested with snow addition at: (**a**) 900 MPa/3 °C, (**c**) 900 MPa/−35 °C and (**e**) 1500 MPa/−15 °C. Area EDS analyses of the corresponding oxide flakes: (**b**) 900 MPa/3 °C, (**d**) 900 MPa/−35 °C and (**f**) 1500 MPa/−15 °C.

As similar tribological conditions exist on the pins and the discs observed in Fig. 2, the friction and wear mechanisms were analysed based on observation of the pins. Figure 3 presents the SEM micrographs of typical worn surfaces on the pins tested with snow. A large area of continuous blackish phase can be observed at the contact path in the tests at all temperatures and both contact pressures (Fig. 3a,c,e). Area EDS analysis was conducted to identify the chemical composition of the blackish phase, and the results are shown in Fig. 3b,d,f.

The EDS results show that the blackish phase is mainly composed of Fe and O with an atomic ratio between iron and oxygen of around 2:3. The blackish phase is therefore deduced to be hematite (Fe_2_O_3_), which is prone to generate in an air-rich, damp environment^23^. Lari *et al.* demonstrated the formation of large platelets of oxide layers under wet conditions^24^. As the pins and discs used in the experiment were carefully cleaned before tests commenced, the hematite is thought to self-generate during the tribological process. Normally, worn features containing large amounts of self-generated oxides in flake form indicate mild wear. This conclusion is based on Quinn’s research, which showed the relationship between wear regimes and the formation of oxides^25^. Lyu *et al.* verified this by achieving a reduced level of wear after oxide flakes self-produced in a simulated wheel-rail contact using a pin-on-disc tribometer^13^. Oxide flakes can self-generate in a rolling-sliding contact as easily as in a pure sliding contact, decreasing the wear between the contacting steels^26^.

Friction, like wear, strongly depends on the oxides at the contacting surfaces. The lubrication effect of the oxide layers can be attributed to their physical properties. Hematite (800 to 1000 HV^27^) is much harder than the base steel (pearlitic steel 500 to 700 HV^28^) and allows only limited elastic/plastic deformation. These characteristics enable hematite to hinder the adhesion between two contacting steel surfaces, and thus abrasion is also reduced. The formation of hematite flakes is likely to be the main reason why the friction coefficient and wear rate were greatly reduced after snow particles were added into the contact. Zhu *et al.* reported a lower friction level because of the formation of oxide flakes with a pin-on-disc tribometer mounted in damp environment^14^.

**Figure 4:**
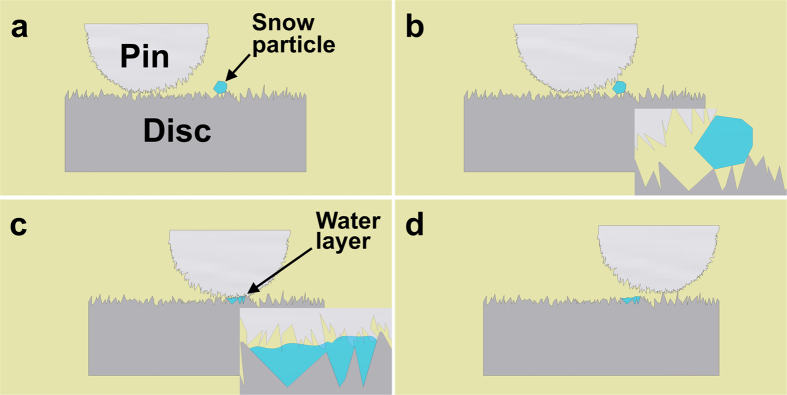
Schematics showing the changing of snow particles into a water layer according to the pressure melting theory.

Although on the macro scale the surfaces of the pin and the disc seem to be as smooth as at manufacture, countless asperities are visible on a micro scale (Fig. 4). Most of the asperities on two shearing surfaces are not homogenous but keep coming in and out of contact. Although the nominal contact pressures between the pin and disc are 900 MPa/1500 MPa, the actual instantaneous pressure on some specific asperities can be much higher. The snow particles added in to the contact must sooner or later encounter these asperities. According to the pressure melting theory of ice/snow (Fig. 4), snow particles melt into a very thin liquid-like layer (Fig. 4c) and adhere to the contacting surfaces under such high pressure^29^. It is feasible to apply this theory to the current test since several researchers have demonstrated in both computational and experimental studies that ice/snow particles exhibit surface melting under high pressure^30^,^31^,^32^,^33^,^34^. When snow is added to the pin and disc contact, the contact is thus thought to contain both air and water. Such an environment is the seedbed for the generation of oxides at the contacting surfaces, consistent with the existence of hematite flakes (Fig. 3). This oxidation reduced the friction and wear after snow particles were added in to the contact. There is no other comparable published information about the influence of snow on the tribology at the wheel-rail contact.

As the snow particles are believed to melt into a water layer due to pressure melting, studies on the effect of water will be discussed. Several laboratory experiments have shown a decrease in the friction coefficient and wear rate with the addition of water to the wheel-rail contact. In these studies, large-scale oxide layers were observed on the worn surfaces^35^,^36^,^37^. A real-world measurement with a hand-pushed tribometer also observed a 0.1 decline in the friction coefficient after water was added to the rail head^38^.

The only research into the effect of snow particles on tribology was carried out with a cast-iron block brake and steel wheel as contacting materials^39^. The friction coefficient decreased about 0.2 after snow particles were applied to the contact. All the above research results are consistent with the findings of the current research, which directly and indirectly verifies the lubrication effect of snow particles at the wheel-rail contact.

### Tribological mechanism at **−**5 °C and **−**15 °C without snow

**Figure 5:**
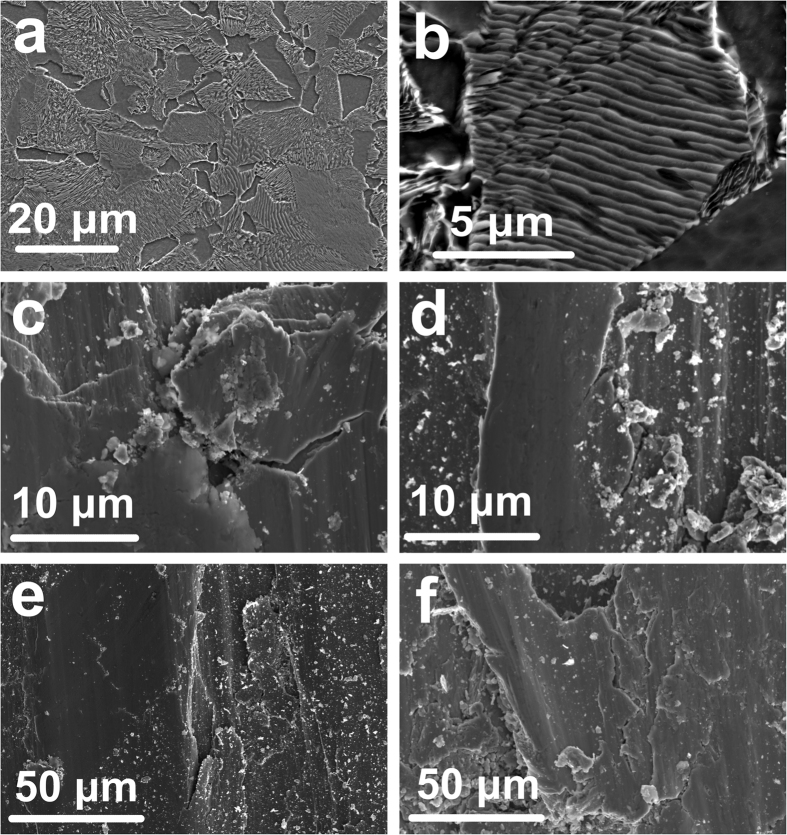
SEM observation of (**a**) typical microstructure of UIC60 900A rail steel and (**b**) a phase boundary between pearlite and ferrite. Cracks on the worn surfaces of pins tested without added snow at (**c**) 900 MPa/−15 °C (the crack is likely to extend along the pearlite-ferrite phase boundary), (**d**) 900 MPa/−5 °C, (**e**) 1500 MPa/−15 °C and (**f**) 1500 MPa/−5 °C.

**Figure 6:**
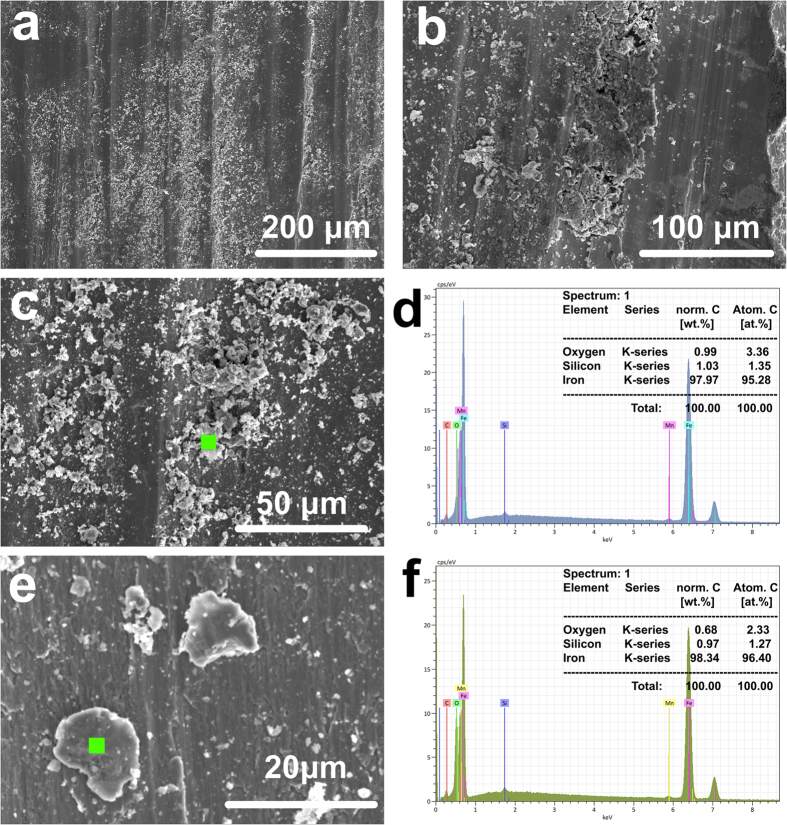
Macroscopic morphology of the wear debris on the worn surfaces of the pins tested without snow addition at: (**a**) 900 MPa/−5 °C and (**b**) 1500 MPa/−15 °C. Microscopic morphology and EDS analyses of the wear debris: (**c**,**d**) 900 MPa/−5 °C and (**e**,**f**) 1500 MPa/−15 °C.

Both the rail and wheel materials used in the current research are pearlitic steels. The typical microstructure of pearlitic steel consists of ferrite and pearlite. Figure 5a shows the morphology of UIC60 900A pearlitic rail steel (pin materials) taken with SEM. The massive grey phase is ferrite and the white lamellar phase is pearlite. There is always a distinguishable phase boundary between the ferrite and pearlite (Fig. 5b). Normally, the ferrite-pearlite phase boundary is enriched by inclusions (such as MnS and oxides) that generate during solidification^40^,^41^. The inclusions give rise to weakness at the phase boundary, which is prone to generate cracks and corrosion pits. There is also a difference in the thermal expansion coefficient between the pearlite and ferrite^42^. Therefore, cracks tend to generate between the ferrite-pearlite phase boundaries at low temperatures due to the low temperature brittleness. Figure 5c shows a typical crack along a pearlite-ferrite phase boundary on the worn surface of a pin tested at −15 °C and 900 MPa without added snow. Similar cracks are common on the worn surfaces of pins tested at −5 °C and −15 °C without snow addition (Fig. 5c–f). Close to the cracks, countless pieces of wear debris can also be observed (bright areas in Fig. 5c–f). At temperatures of −5 °C and −15 °C without snow, all the worn surfaces are covered by debris and a large number of scratching features are present (Fig. 6a,b).

Point EDS analyses were conducted to identify the composition of the wear debris, and the results are shown in Fig. 6c–f. The wear debris is almost pure iron (about 98 wt.% as shown in Fig. 6d–f). It can be concluded that the wear debris originated from the base metal and resulted in the scratching features shown in Fig. 6a,b during sliding. The most likely origin of the wear debris is the bulk scraps generated due to low temperature brittleness. In this process cracks were generated along the ferrite-pearlite phase boundary. With repeated shearing, these cracks gradually extended and resulted in the spalling of bulk scraps, which were subsequently involved in the sliding process as a third-body phase. The bulk scraps were also susceptible to low temperature brittleness and were easily crushed into tiny wear debris under continuous shearing between the pin and disc.

Oxidation seldom occurred in the tests without snow at −5 °C and −15 °C, as shown by the fact that neither oxide flakes nor oxide pits were found on the worn surfaces (Fig. 6a,b). The tribological mechanism in tests without snow at −5 °C and −15 °C is likely to be abrasion/ploughing and differs significantly from the tests with snow. Abrasion/ploughing was widely found at the contact containing high hardness third-body debris. With graphene added into an alumina composite, higher wear rate and scratching features were found on the mating surfaces^7^,^43^. In previous tribological tests with steel^44^ and brass^45^ samples, friction and wear increased when hard asperities became embedded in the soft base surface, which increased the abrasion/ploughing effect. In wheel-rail contacts, typical third-body debris involves sands and oxide particles, both of which have been demonstrated to easily embed in wheel and rail steels. This increases the friction coefficient and wear rate through the abrasion/ploughing mechanism. In a laboratory-scale test with a twin-disc apparatus, the friction coefficient increased 0.2 when sand was added into the wheel-rail contact, and the wear rate increased more than 3 times^11^. Another laboratory experiment using a twin-disc setup showed similar results with the wheel-rail contact with sand addition showing a 0.2 increase in the friction coefficient compared with the contact without sand addition^37^. Oxide particles have also been shown to increase the friction coefficient and wear rate at the wheel-rail contact where abrasive features were found on the worn surfaces^10^,^13^.

### Ice condensation at temperatures below **−**15 °C

**Figure 7:**
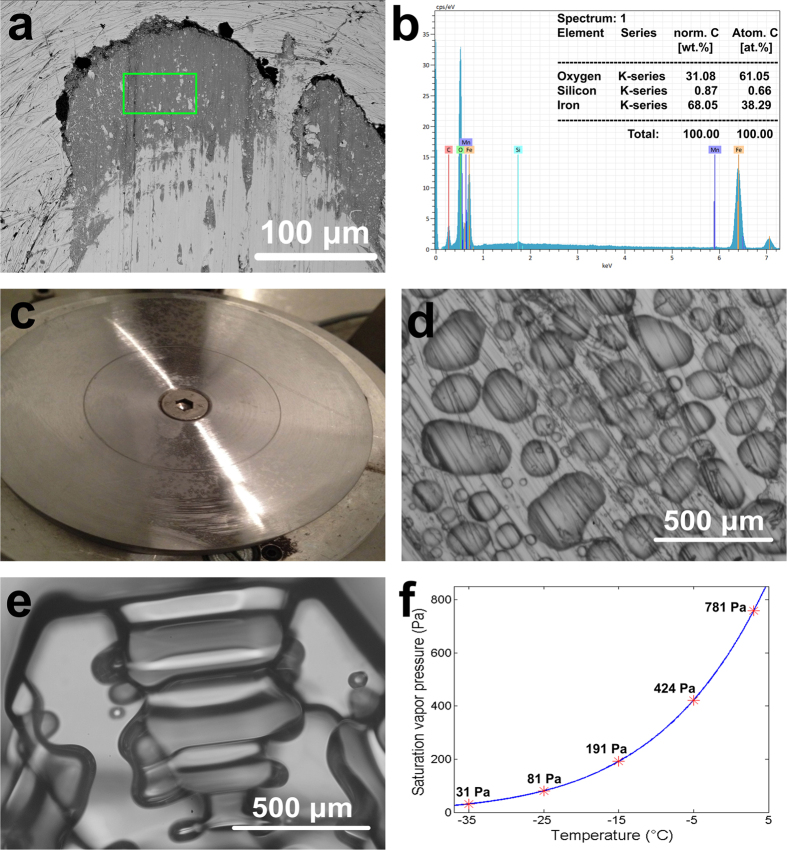
An explanation of low friction coefficient and wear rate below −15 °C without snow addition. (**a**) Oxide flake on the worn surface of pin tested at 1500 MPa/−35 °C. (**b**) area EDS analysis of the oxide flake shown in (**a**). (**c**) Macroscopic and (**d**) microscopic morphology of the ice layer condensed on the disc at 1500 MPa/−35 °C. (**e**) Morphology of a natural hexagonal snow particle. (**f**) Super-saturation vapour pressure as a function of temperature^46^.

The friction coefficient and wear rate in tests without snow addition increase noticeably as the temperature drops from 3 °C to −15 °C. By contrast, when the temperature drops from −15 °C to −35 °C (Fig. 2) the friction coefficient and wear rate decrease sharply, a change that cannot be attributed to the ductile-brittle transition of steel. The worn surface of the pin tested at −35 °C and 1500 MPa without snow was tested using SEM to determine the tribological mechanism, and the results are shown in Fig. 7a. A large area of blackish phase can be observed at the contact path, an observation that is very similar to that for tests with snow (Fig. 3a,c,e). EDS analysis confirmed that the deposit is hematite (Fe_2_O_3_) as the atomic ratio between iron and oxygen is about 2:3 (Fig. 7b). The same oxide flakes were generated here as in the tests with snow, which explains the sharp decrease in the friction coefficient and wear rate when the temperature decreased from −15 °C to −35 °C (Fig. 2). It is suspected that some agent is generated during the tests which encourages the generation of oxide flakes, as hematite is prone to form in humid air.

Figure 7c shows the disc surface after testing without snow addition at −35 °C. A large-scale ice layer has condensed on the disc surface. The microscopic morphology of the condensed ice layer measured *in-situ* with optical microscopy (OM) is shown in Fig. 7d. Compared with the natural snow particle observed *in-situ* (Fig. 7e), the condensed ice particles are smaller in size and have an irregular structure. Although the ice particles condensed on the disc surface discontinuously, they were thought to participate in the tribological process in the same way as the snow particles added intentionally. Their presence encourages the generation of oxide flakes in the contact path, which in turn reduces the friction coefficient and wear rate at the wheel-rail contact.

To confirm the possibility of ice condensation on the disc surface, the saturation vapour pressure as a function of temperature in the range used in the experiment was calculated according to Wexler’s equation (1)^46^:


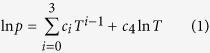


where *T* is the temperature in Kelvin and *c*_*0*_ to *c*_*4*_ are fitting coefficients (*c*_*0*_ = −0.60436117 × 10^4^, *c*_*1*_ = 0.1893292601 × 10^2^, *c*_*2*_ = −0.28244925 × 10^−1^, *c*_*3*_ = 0.17250331 × 10^−4^, *c*_*4*_ = 0.2858487 × 10). Figure 7f plots this equation, the correlation between the saturation vapour pressure (Pa) and the temperature (°C). The corresponding saturation vapour pressure values at the temperatures tested are indicated by asterisks. It can be seen that the air becomes saturated more easily at lower temperatures (at −35 °C the saturation vapour pressure is one order of magnitude lower than that at 3 °C). Kuroda *et al.* discuss the kinetics of the ice condensation process, demonstrating that the ice condensation rate is a decreasing function of the saturation vapour pressure^47^. The above two scenarios jointly show the possibility of an ice condensation layer on the metal surfaces (Fig. 7c,d) when temperature decreased to −35 °C. This condensed ice layer would act in the same way as the snow particles added intentionally, and would significantly reduce the friction coefficient and wear rate at the wheel-rail contact.

## Conclusions

The tribological performance at the wheel-rail contact in the open system (different temperatures from −35 °C to 3 °C, with and without added snow) was investigated using a pin-on-disc tribometer mounted in a temperature-controlled environmental chamber. It was found that the friction coefficient and wear rate are much more sensitive to temperatures in this range than at room temperatures.

The snow particles added to the wheel-rail contact melt into a liquid-like layer during sliding because of pressure melting and encourage the formation of hematite (Fe_2_O_3_) flakes at the contact, dramatically reducing the friction and wear. Once there are snow particles in the contact, the wear rate becomes independent of temperature because oxidative wear governs the mild wear process.

In the absence of snow, the tribological process is controlled by the low temperature brittleness in the temperature range from 3 °C to −15 °C. Cracks are prone to form on the worn surfaces, which extend gradually and result in the spalling of bulk scraps. The bulk scraps are subsequently crushed into tiny debris that sharply increase the friction coefficient and wear rate due to strong abrasion. When the temperature decreases to −25 °C, an ice layer condenses on the metal surfaces and has a similar effect on the tribological process to that of the added snow particles. The ice layer also causes hematite (Fe_2_O_3_) to form flakes that decreases the friction coefficient and wear rate.

## Materials and Methods

### Materials

The materials used in the research were cut from R7 wheel and UIC60 900A rail steels, both of which are commonly used wheel and rail materials. The chemical composition (wt.%) for the R7 wheel is 0.7% C, 0.3% Si, 1.0% Mn, 0.04% P, 0.04% S. For the UIC60 900A rail the composition is 0.52% C, 0.4% Si, 0.8% Mn, 0.035% P, 0.035% S.

### Impact toughness measurement

The impact toughness was measured according to a standard method for metallic materials, ISO 148-1 (International Standard for Metallic Materials-Charpy Pendulum Impact-Test Part 1: Test Method). Specimens for the impact toughness measurement were manufactured from UIC60 900A rail steels by wire cutting into a cuboid shape (10 × 10 × 55 mm^3^), containing a V-notch of 2 mm in depth with a 45° angle. A JBDS-300C digital low temperature Charpy pendulum impactor was applied at testing temperatures of 20 °C, 3 °C, −5 °C, −15 °C, −25 °C, −35 °C, respectively. Tests were repeated three times at each temperature level.

### Tribology testing technique

**Figure 8:**
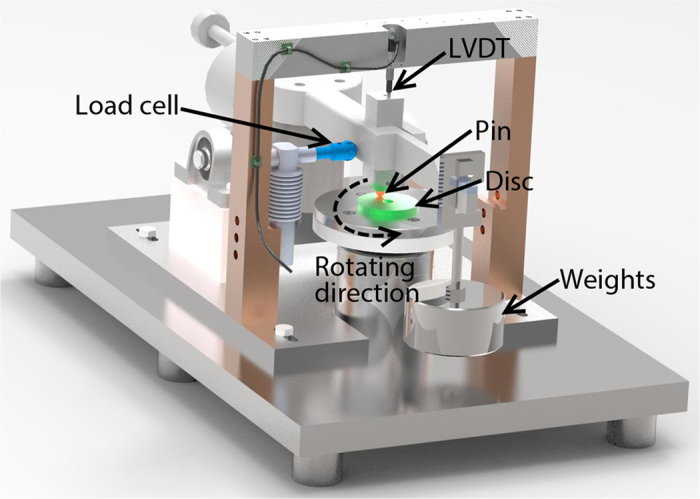
Pin-on-disc tribometer.

The friction and wear tests were conducted using a pin-on-disc tribometer that contained a horizontally rotating disc and a dead-loaded pin (Fig. 8). A group of weights connecting the fulcrum through a beam provide the normal force at the contact between disc and pin. The rotation rate is controllable so that a pure sliding contact is achievable at various speeds between the pin and disc.

The discs were manufactured from R7 wheel and pins from UIC60 900A rail steel. The pins featured a uniform tip radius of 5 mm and the discs (50 mm in radius) were flat. Accurate grinding was performed so that both the pin and disc surfaces had a centre-line-average roughness of 0.6 μm, which is common on railway wheels and rails^48^. Before testing commenced, all the samples were subjected to a standard cleaning procedure: 10 min ultrasonic cleaning in heptane and methanol, respectively, followed by oven-drying at 100 °C for 20 min.

The sliding speed was fixed at 0.01 m s^−1^. Two contact pressures were chosen, 900 MPa and 1500 MPa (calculated using Hertzian contact theory^49^), This combination of sliding speed and contact pressures represents typical rail head-wheel tread contact conditions^50^. The tests were carried out in a quasi-sealed, temperature-controlled environmental chamber at temperatures of 3 °C, −5 °C, −15 °C, −25 °C, −35 °C. Half of the tests were conducted with the addition of natural fresh snow particles, collected as needed. The snow particles were added to the pin-disc contact every 5 min with a 17 ml spoon. Every test condition was repeated three times. Each test lasted 30 min (a sliding distance of 18 m). The friction force was measured by a HBM Z6 load cell throughout the tests. The friction coefficient was calculated by dividing the friction force by the normal force. An average for each test was calculated using the friction data from the last 10 min to exclude the running-in phase. Mean values and standard deviations were the final form of the friction results. These values were calculated from the averages of the three repeats within each test condition.

The wear volume of the pin was calculated based on the equation for an ellipsoid segment after measuring the wear scar with a Nikon MM-60 measuring microscope. The specific wear rate was calculated using Archard’s equation (2)^43^:


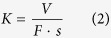


where *K* is the wear rate, *V* the wear volume, *F* the normal force and *s* the sliding distance. The wear rate results were also in the form of mean values and standard deviations for the three repeats within each test condition.

### Characterisation

Features of the worn surface were observed using SEM (Hitachi S-3700N). Chemical compositions at specific areas were measured by EDS (Bruker XFlash 6–10). The microstructure of specimens was examined using SEM (JSM-7800F Field Emission Scanning Electron Microscope). The imaged surface was first ground with 400 grit, 800 grit and 1200 grit sandpaper and then polished and etched with 4% nital for 30 sec. The cross-sectional wear tracks on the disc samples were measured using a Talysurf PGI 800 (Taylor/Hobson Precision, UK) with a stylus tip radius of 2 μm. The examined distance was 4 mm and the gap between each sample point was 5 μm. The morphology of the collected snow particles and condensed ice layers on the disc surface were observed using an OM (Olympus BH2 System Microscope).

## Additional Information

**How to cite this article**: Lyu, Y. *et al.* Open System Tribology and Influence of Weather Condition. *Sci. Rep.* **6**, 32455; doi: 10.1038/srep32455 (2016).

## Figures and Tables

**Figure 1:**
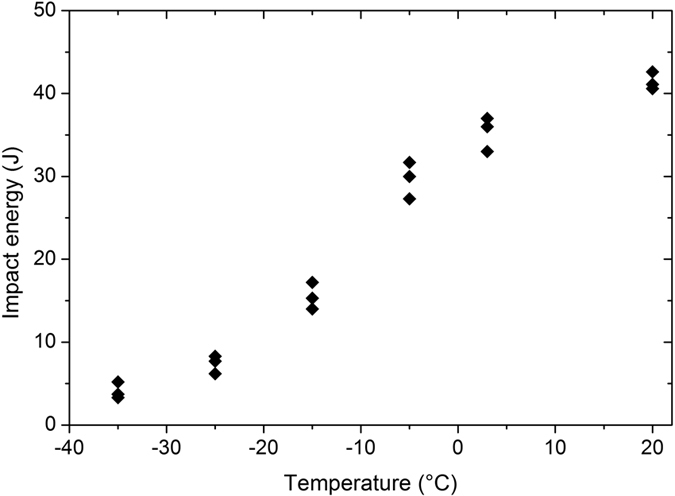
Impact energy of UIC60 900A rail steel as a function of temperature, showing a ductile-brittle transition with temperature decrease.
